# Construction of Antifouling Membrane Surfaces through Layer-by-Layer Self-Assembly of Lignosulfonate and Polyethyleneimine

**DOI:** 10.3390/polym11111782

**Published:** 2019-10-31

**Authors:** Lin Gu, Meng-Yun Xie, Yu Jin, Min He, Xiao-Yan Xing, Yuan Yu, Qing-Yun Wu

**Affiliations:** 1 School of Chemical Engineering and Technology, Sun Yat-sen University, Zhuhai 519082, China; gulin1985@gmail.com; 2 Faculty of Materials Science and Chemical Engineering, Ningbo University, Ningbo 315211, China; xmy1993_1005@163.com (M.-Y.X.); cathystang@163.com (Y.J.); amtalya11@gmail.com (M.H.); xxy18755023221@163.com (X.-Y.X.); yuyuan891226@163.com (Y.Y.)

**Keywords:** lignin, polysulfone membrane, layer-by-layer self-assembly, antifouling, surface modification, hydrophilic

## Abstract

Lignin is the second most abundant and low-cost natural polymer, but its high value-added utilization is still lack of effective and economic ways. In this paper, waste lignosulfonate (LS) was introduced to fabricate antifouling membrane surfaces via layer-by-layer self-assembly with polyethyleneimine (PEI). The LS/PEI multilayers were successfully deposited on the polysulfone (PSf) membrane, as demonstrated by ATR-FTIR, XPS, Zeta potential measurements, AFM, and SEM. Meanwhile, the effect of the number of bilayers was investigated in detail on the composition, morphologies, hydrophilicity, and antifouling performance of the membrane surface. As a result, with the bilayer numbers increase to 5, the PSf membrane shows smooth surface with small roughness, and its water contact angle reduces to 44.1°, indicating the improved hydrophilicity. Accordingly, the modified PSf membrane with 5 LS/PEI bilayers repels the adsorption of protein, resulting in good antifouling performance. This work provides a green, facile, and low-cost strategy to construct antifouling membrane surfaces.

## 1. Introduction

Water scarcity and pollution have been considered as the two main environmental problems of global concern [1]. In the past few decades, membrane technology has been widely developed for water purification with low cost and energy [1,2,3]. Polymeric membranes are most commonly employed for water treatment, due to their easy scale-up, high water quality, and excellent separation efficiency [4,5]. Among others, polysulfone (PSf) has been widely used to fabricate various membranes, such as microfiltration (MF), ultrafiltration (UF), and nanofiltration (NF) membranes, due to its excellent mechanical properties and superior acid and alkali resistance [6,7,8]. However, the hydrophobicity of PSf causes membrane fouling, which is considered as a bottleneck, limiting its wide spread applications [7,8]. Many investigations have illustrated that surface hydrophilic modification is the most common method to effectively reduce the PSf membrane fouling. This is because the hydrophilic chains can form a tight hydration layer on the membrane surface and then repel foulants adsorption via repulsive hydration forces [6,9,10,11]. Although various materials and fabrication approaches have been reported for surface modification, more efforts should be made to develop a green, efficient, and lost-cost strategy [12,13,14].

Layer-by-layer (LbL) assembly has been proven to be a versatile and facile approach to construct very thin polyelectrolyte multilayer films, making it highly suitable for membrane surface modification [15,16,17]. In generally, these multilayer films are easily produced by consecutive adsorption of alternating anionic polyelectrolyte and cationic polyelelctrolyte through electrostatic interaction. The thickness, the surface morphology, and properties of the multilayer can be modulated by controlling the repeating adsorption times, building species, and adsorption circumstances. Moreover, compared with conventional surface modification [18], LbL assembly technology could effectively alleviate the hydraulic resistance increase and water flux decrease by controlling thickness of the extra layer [16]. A variety of polyelectrolytes have been applied as build substances to construct antifouling multilayer films on membrane surfaces [19,20,21,22,23]. For example, poly(sodium 4-styrene sulfonate)/poly(diallyldimethylammonium chloride) films were formed on an anion exchange membrane to generate a hydrophilic and negatively charged surface layer, improving antifouling performance to organic foulant in electrodialysis [20]. However, most of these polyelectrolytes have complex molecular structures and are always synthesized through complicated and time-consuming processes, which offset the facility of LbL assembly to some extent.

**Figure 1:**
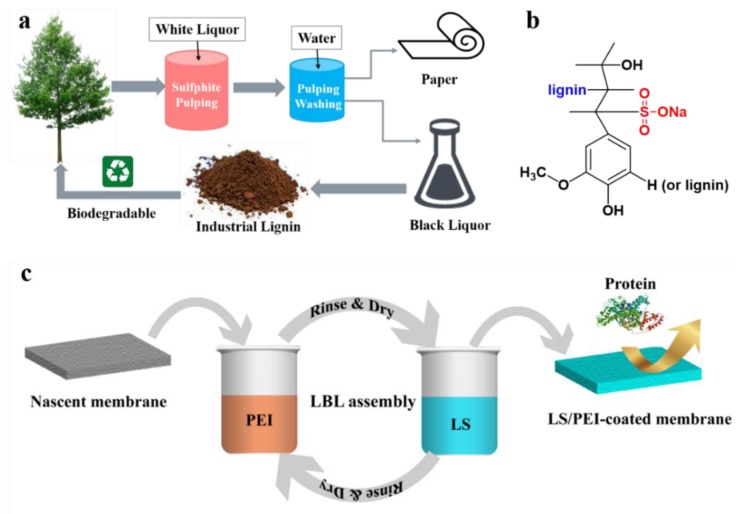
(**a**) Schematic diagram for chemical pulp production; (**b**) Structural diagram of sodium lignosulfonate (LS); (**c**) Schematic illustration of the LbL deposition of sodium lignosulfonate and polyethyleneimine (LS/PEI) for antifouling membrane surfaces.

Nowadays, great attention has been paid to renewable and biodegradable resources due to the rapid consumption of fossil fuels and growing environment pollution [24,25,26]. As a primary constitute (15%–40%) of lignocellulosic biomass, lignin is the second most abundant and relatively inexpensive natural polymer [27,28,29,30,31]. Approximately 70 million tons of industrial lignin (e.g., lignosulfonate, Kraft lignin) are produced each year during the extraction of cellulose for the pulp and paper industry (Figure 1a) [29,31,32]. Among others, lignosulfonate (LS), as a byproduct recovered from a sulfite pulping process, is a naturally water-soluble anionic polyelectrolyte (Figure 1b), which could be used to fabricate LbL multilayers via electrostatic interaction [33,34]. Regarding the molecule itself, LS not only has no toxicity and good biodegradability, but also possesses numerous functional groups such as phenol, hydroxyl, and carboxyl, making it a promising building-block in sustainable materials [29,31,35]. The assembly of LS on the substrates can be used for surface modification [36,37,38,39,40], metal ion adsorption [41], and even as a flame retardant [25,42]. For example, Li et al. [34] chose LS as the anionic polyelectrolyte and chitosan (CS) as the cationic polyelectrolyte to modify the surfaces of cellulose fibers through LbL deposition technique. The obtained multilayers showed good antimicrobial and antioxidant properties. However, the surface modification of LS multilayers is by far focused on fibers, nanoparticles, and paper, which strongly limit the applications of the LS-based LbL assembly multilayers.

In this work, waste LS was introduced to construct antifouling membrane surfaces via LbL assembly (Figure 1c). The LS/polyethyleneimine (PEI)-coated PSf membranes were characterized by ATR-FTIR, XPS, Zeta potential measurements, AFM, and SEM. Moreover, the effect of the number of bilayers on the composition, morphologies, hydrophilicity, and antifouling performance of the modified membrane surfaces were investigated in detail. Overall, this work indeed provides a green, facile, and low-cost strategy to construct antifouling membrane surfaces.

## 2. Experimental

### 2.1. Materials

Polysulfone (PSf, *M*_n_ = 22,000) was supplied by Solvey Co., Shanghai, China and dried at 60 °C in vacuum before use. Sodium lignosulfonates (LS) were kindly provided by Shandong Wei Li Corporation, Shandong, China. Polyethyleneimine (PEI, *M*_w_ = 70,000) was purchased from Aladdin Industrial Corporation, Shanghai, China. Polyethylene glycol (PEG, *M*_n_ = 400) and N, N-dimethylacetamide (DMAc, 99%) were commercially supplied by Sinopharm Chemical Reagent Co., Ltd., Shanghai, China. Bovine serum albumin (BSA) was obtained from Solarbio Technology Co., Ltd., Beijing, China.

### 2.2. Fabrication and Characterization of LS/PEI Multilayers on PSf Membranes

#### 2.2.1. Preparation of PSf Membranes

PSf membranes were prepared by nonsolvent induced phase separation method according to our previous work [30,43]. PSf (4.41 g) and PEG (1.47 g) were dissolved in DMAc (23.30 g) at 60 °C for 8 h. After degassing, the mixed solution was cast on a glass plate by using a casting knife with a thickness of 200 μm, and then immersed into a water bath containing 0.3% (*v*/*v*) DMAc to initiate a phase inversion. Herein, DMAc in the water bath was used to obtain porous membrane surfaces by delaying the phase separation process. The obtained PSf membrane was transferred to another water bath and stored for at least 1 day to remove the residual solvent.

#### 2.2.2. LbL Deposition

The LbL deposition process is presented in Figure 1c. The PSf membrane was first immersed in PEI aqueous solution (0.1 g/L) for 15 min, rinsed thoroughly using deionized (DI) water to remove excess PEI solution, and then immersed in DI water for 15 min. Subsequently, the PSf membrane with PEI molecules was immersed into 1 wt % LS aqueous solution (pH = 8) for 15 min, following the same rinsing to remove excess LS solution. One bilayer was built up by a PEI layer and a LS layer. After the desired number of the bilayers was reached, the sample was dried in vacuum and then stored in a desiccator. The PSf membrane coated with LS/PEI multilayers was named as PSf-xBL, where x indicates the number of the bilayers.

#### 2.2.3. Characterization

Attenuated total reflection Fourier transform infrared (ATR-FTIR) spectra were obtained on a Nicolet 460 (Thermo Nicolet Corporation, Shanghai, China) using 64 scans at a spectral resolution of 2 cm^−1^. A differential spectrum was obtained by using OPUS spectral analysis software (Bruker Scientific Technology Co., Ltd., Beijing, China). X-ray photoelectron spectroscopy (XPS) measurements were performed on a Kratos Axis Ultra spectrometer (Axis Ultra DLD, Kratos Analytical Ltd., Manchester, UK). Surface zeta potential measurements were conducted on a SurPASS zeta potential analyzer (Anton Paar GmbH, Shanghai, China). Field emission scanning electron microscopy (FESEM) was carried out on a FEI S-4800 scanning electron microscope (Hitachi, Tokyo, Japan). Atomic force microscopy (AFM) was observed on a Veeco 3100 scanning probe microscope (Veeco, Shanghai, China). Static contact angles of water droplets on the membrane surfaces were obtained using a DSA109 optical contact angle measurement system (KRUSS GmbH, Hamburg, Germany).

### 2.3. Antifouling Performances of the Membranes

#### 2.3.1. Static Protein Adsorption

BSA was chosen as the model foulant, and the membranes were immersed in a BSA solution of PBS (1.0 g/L) for 2 h at room temperature under neutral pH. A standard curve of absorbance-BSA concentration was obtained from UV absorbance at 280 nm using a TU1901 UV–vis spectrophotometer (Beijing Purkinje General Instruments Co., Ltd., Beijing, China) (see Figure S1). The BSA adsorption capacities of the membranes were estimated by calculating the concentrations difference of BSA solution before and after adsorption.

#### 2.3.2. Dynamic Antifouling Test

Dynamic antifouling test was carried out by a dead-end filtration system (Millipore Corporation, Billerica, MA, US). Firstly, deionized water was permeated as a feed at 0.12 MPa for 30 min and the pure water flux (*J*_0_) was obtained. Secondly, BSA solution (1 g/L) under neutral pH was filtrated and the water flux of BSA solution (*J*_1_) was measured. Thirdly, PBS solution was used in hydraulic washing for 30 min. Deionized water was permeated again as a feed and another pure water flux (*J*_2_) was determined. The flux recovery ratio (*FRR*), the total flux decline ratio (*DR*_t_), and irreversible flux decline ratio (*DR*_ir_) can be defined according to the references [1,3,44]:

(1) $$FRR=\frac{J_{2}}{J_{0}}\times 100\%,$$

(2) $$DR_{t}=\frac{J_{0}-J_{1}}{J_{0}}\times 100\%,$$

(3) $$DR_{ir}=\frac{J_{0}-J_{2}}{J_{0}}\times 100\%.$$

## 3. Results and Discussion

### 3.1. Formation of LS/PEI Multilayers on PSf Membranes

**Figure 2:**
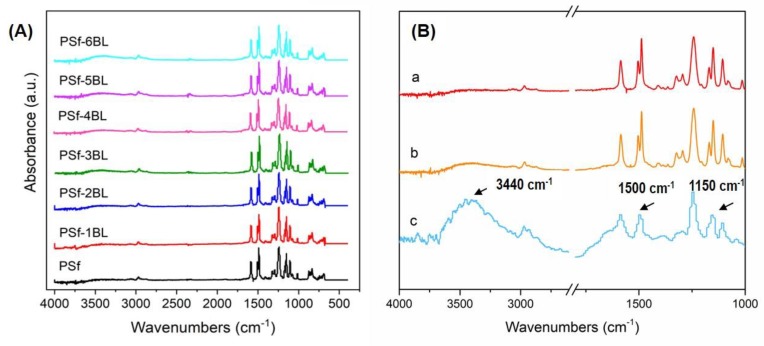
ATR-FTIR spectra of (**A**) PSf membranes with different bilayer numbers, and (**B**) PSf (**a**) and PSf-5BL (**b**) membranes, and their differential spectrum (**c**).

ATR-FTIR and XPS were employed to characterize the surface composition of nascent and modified PSf membranes during the LbL assembly process. The survey ATR-FTIR spectra of nascent and modified membranes are shown in Figure 2. It can be clearly observed that the intensity of a broad peak at ca. 3440 cm^−1^ increases with the increase of the bilayer numbers of LbL assembly, which is ascribed to the stretching vibration of –OH groups of LS and –NH groups of PEI [36,45]. Additionally, in the differential spectrum of modified and nascent PSf membranes (Figure 2B,c), the bands at 1500 and 1150 cm^−1^ are corresponded to the aromatic ring and sulfonic groups (S−O) in LS, respectively [36].

**Figure 3:**
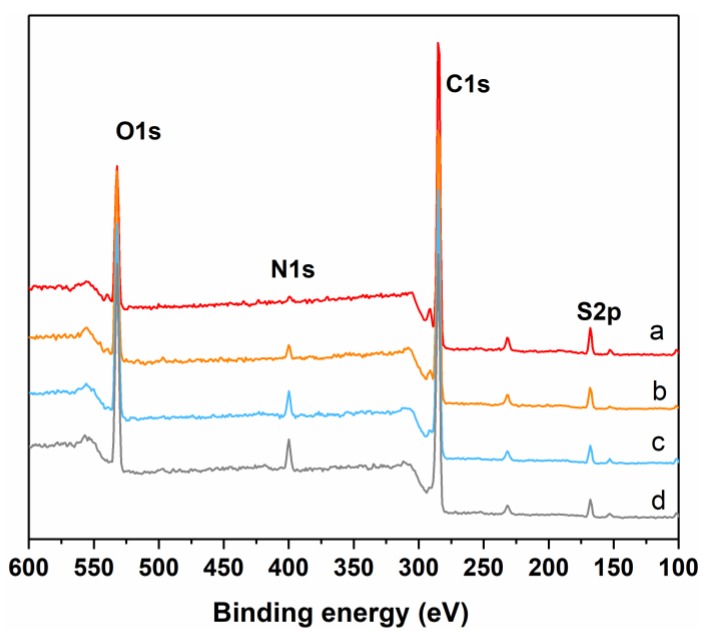
XPS spectra of (**a**) PSf, (**b**) PSf-1BL, (**c**) PSf-3BL, and (**d**) PSf-5BL membranes.

Figure 3 shows the XPS spectra of nascent and modified PSf membranes. The nascent membrane displays three distinctive peaks at 167.7 eV, 284.6 eV, and 532.8 eV, which are attributed to the binding energy of S2p, C1s, and O1s, respectively. For modified membranes, the distinctive peak at 399.3 eV from N1s indicates the existence of PEI. Besides, the intensity of the N1s peak is gradually enhanced with the increasing bilayer numbers (Table 1). Both ATR-FTIR and XPS demonstrate that LS/PEI multilayers are successfully deposited on the PSf membrane surfaces via LbL assembly.

**Figure 4:**
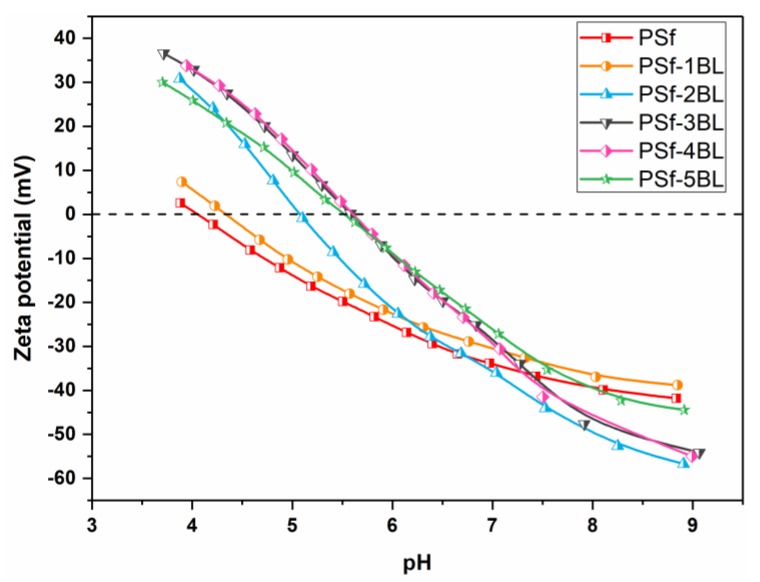
Plots of Zeta potential of nascent and modified PSf membranes versus pH.

The surface charge of nascent and modified PSf membranes was monitored using surface zeta potential measurements. The zeta potential of nascent and modified membranes under different pH values is presented in Figure 4. The nascent membrane has a negative potential under pH > 4. Hence, PEI with positive charge could be easily deposited on the membrane surface as the first layer, and subsequent deposition of LS onto PSf membrane remains a negative potential under pH > 4.5. More negative potential under pH > 8 was observed with the further deposition of PEI and LS, suggesting that the deposition process of PEI and LS on the membrane surface could be realized in a reproducible way.

### 3.2. Surface Morphologies of LS/PEI Multilayers on PSf Membranes

**Figure 5:**
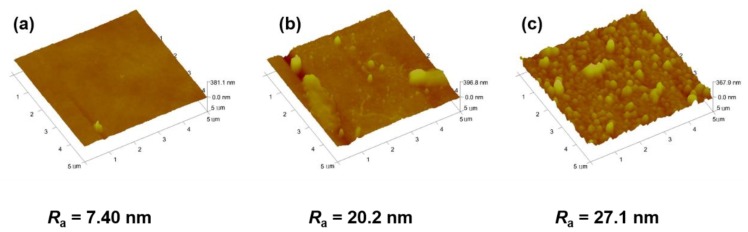
AFM images of PSf-1BLmembranes prepared from different pH values of LS aqueous solution. (**a**) pH = 8; (**b**) pH = 7; (**c**) pH = 5.

It is well known that the pH value could affect the aggregation behavior of polyelectrolytes [39]. The adsorption of the first LS layer onto PSf membrane was conducted at pH 8, 7, and 5, respectively. When prepared at pH 8, the PSf-1BL membrane shows a smooth surface (Figure 5a), while the surfaces of the modified membranes become rough and exhibit granular LS aggregate at pH 7 and 5 (Figure 5b,c). The surface roughness (*R*_a_) in Figure 5a is 7.40 nm, much lower than 20.2 nm in Figure 5b or 27.1 nm in Figure 5c. This is because LS is sterically stabilized in base solutions while LS tends to aggregate in acidic solutions, which has been reported by Nyman et al. [46]. In addition, the LS/PEI multilayers on PSf membranes prepared at pH 5 and 7 are unstable in the process of water filtration (see Figure S2). In this work, the modified PSf membranes were prepared from LS solution at pH 8 unless otherwise specified.

**Figure 6:**
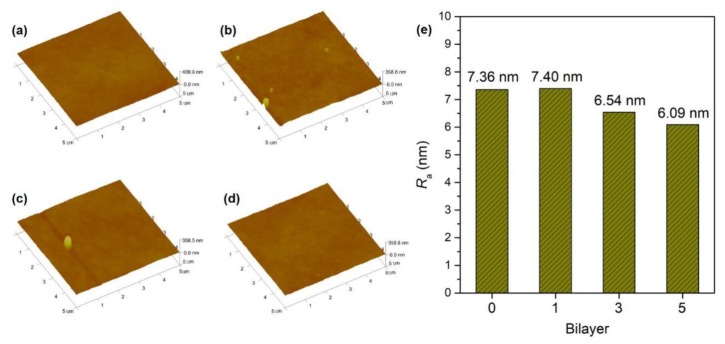
AFM images of PSf (**a**), PSf-1BL (**b**), PSf-3BL (**c**), and PSf-5BL (**d**) membranes, and (**e**) the corresponding *R*_a_ of membranes with different bilayer numbers.

**Figure 7:**
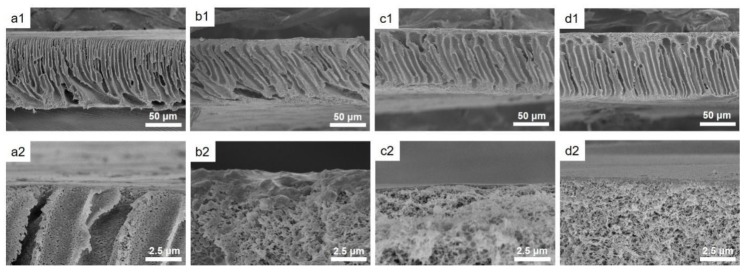
SEM images of cross-section morphologies of PSf (**a1**, **a2**), PSf-1BL (**b1**, **b2**), PSf-3BL (**c1**, **c2**), and PSf-5BL (**d1**, **d2**) membranes.

Figure 6 exhibits the surface morphologies of the modified PSf membranes with different bilayer numbers. The nascent membrane exhibits a smooth surface with a *R*_a_ of 7.36 nm. LbL deposition of LS/PEI multilayers has little effect on the membrane surface, and the *R*_a_ is maintained between 6~7.4 nm. Overall, the modified membranes have relatively smooth surfaces, which is beneficial to fouling resistance [15]. The cross-section morphologies of the PSf membranes modified with LS/PEI multilayers investigated by SEM are shown in Figure 7. It can be seen that the PSf membrane has finger-like pore structure. A thin film can be also observed on the membrane surfaces after magnifying to 10,000 times, and becomes thicker with the increasing bilayer numbers.

### 3.3. Antifouling Performances of the Membranes

**Figure 8:**
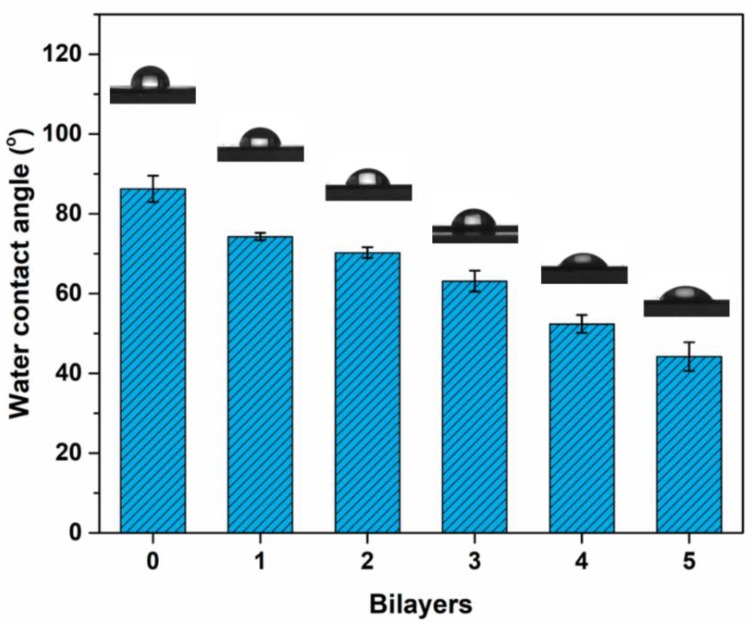
Water contact angles of the PSf membranes with different bilayer numbers.

Generally, the hydrophilic surfaces could easily adsorb water molecules and form a hydration layer, and thus, repel foulant adsorption via repulsive hydrated forces [15]. The surface hydrophilicity of the modified PSf membranes with different bilayer numbers was evaluated by a static water contact angle, as shown in Figure 8. The nascent membrane has a water contact angle of 86.2°. It decreases significantly with the increasing the bilayer numbers. The water contact angle is 44.1° for the PSf-5BL membrane. This result demonstrates that LbL deposition of LS/PEI multilayers can achieve hydrophilic modification of the membranes, which may inhibit the foulant adsorption from solutions.

**Figure 9:**
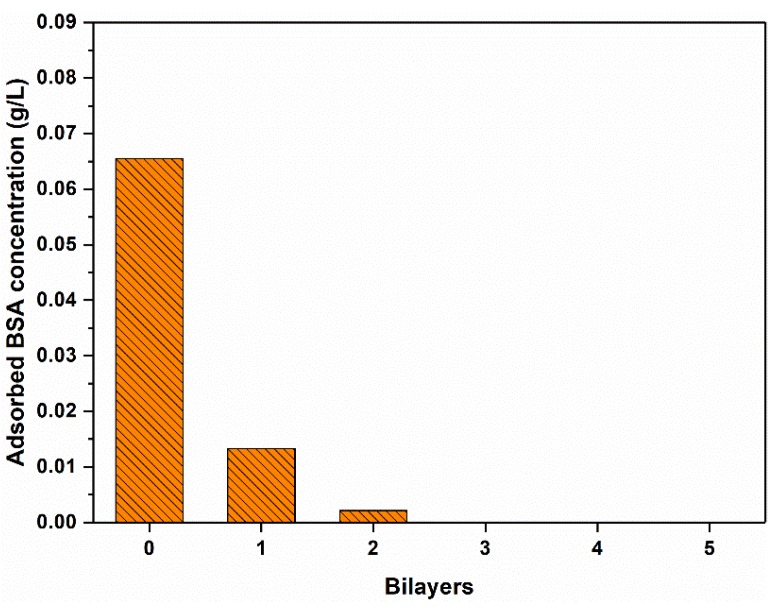
Adsorbed BSA concentration of the PSf membranes with different bilayer numbers under neutral pH.

**Figure 10:**
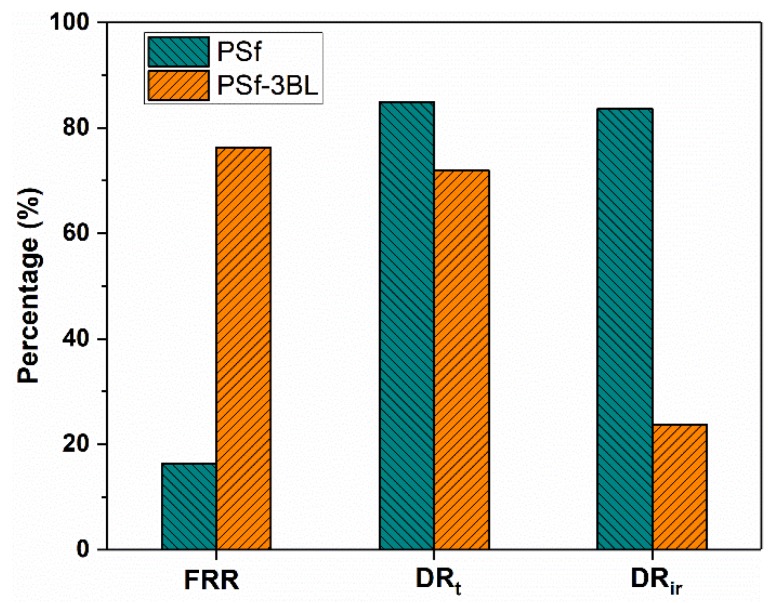
A summary of the corresponding *FRR*, *DR*_t_, and *DR*_ir_ values of PSf and PSf-3BL membranes during the dynamic BSA filtration under neutral pH.

Antifouling performances of the membranes were characterized using BSA as the model foulant under neutral pH. Figure 9 shows adsorbed BSA amount on the nascent and modified PSf membranes. The surfaces of the LS/PEI-modified PSf membranes display significantly low BSA adsorption. When the bilayer number increases to 3, the BSA adsorption amount of the modified membrane almost decreases to 0. The dynamic antifouling performance of the modified PSf membrane has been further characterized (Figure S3). Figure 10 lists the *FRR*, *DR*_t_, and *DR*_ir_ values of the PSf and PSf-3BL membranes. Among them, *DR*_ir_ reflects the irreversible fouling, which is very difficult to be eliminated by the hydraulic washing, and the main challenge in the membrane separation [44,47]. Overall, membranes with lower *DR*_ir_, *DR*_t_, and higher *FRR* are regarded as having better antifouling performance [3,11]. As for the PSf membrane, the *DR*_ir_ and *DR*_t_ are as high as 83.7% and 84.9%, respectively, corresponding to *FRR* as low as 16.3%. However, the value of *DR*_ir_ for the PSf-3BL membrane is only 23.7%, corresponding to 76.3% flux recovery ratio. These results indicate the PSf membranes modified with LS/PEI multilayers have excellent antifouling property.

## 4. Conclusions

The antifouling membrane surfaces were achieved through constructing LS/PEI multilayers on the PSf membrane via LbL assembly. Results from AFM and SEM show the LS/PEI-coated membranes have smooth surfaces and the thickness of the coating increases with the increasing bilayer numbers. The multilayers endow the membranes with good hydrophilicity and excellent antifouling performance. The adsorption amounts of BSA on the modified membrane almost decrease to 0 when the bilayer number reaches to 3. In addition, the modified membranes show larger *FRR*, lower *DR*_t_ and *DR*_ir_ compared to nascent membrane. This work provides a green, facile, and low-cost strategy to construct antifouling membrane surfaces.

## Figures and Tables

**Table 1:** Surface composition of PSf, PSf-1BL, PSf-3BL, and PSf-5BL membranes from XPS spectra (in atomic percent).

Samples	N1s	C1s	O1s	S2p
PSf	−	79.08%	16.24%	4.67%
PSf-1BL	3.03%	72.85%	20.08%	3.65%
PSf-3BL	5.33%	68.98%	21.63%	3.27%
PSf-5BL	6.12%	68.67%	22.12%	3.09%

## Supplementary Materials

The following are available online at https://www.mdpi.com/2073-4360/11/11/1782/s1, Figure S1: A standard curve of UV absorbance-BSA concentration, Figure S2: UV absorbance at 280 nm of aqueous solutions after immersing the PSf-5BL membranes prepared at different pH values for 2 h, Figure S3: Flux of water and BSA: first for pure water, second for BSA and third for pure water after washing the membranes for 30 min.
