# Supplementary material for: Construction of Antifouling Membrane Surfaces through Layer-by-Layer Self-Assembly of Lignosulfonate and Polyethyleneimine
Source: Polymers (Basel). 2019 Oct 31;11(11):1782. doi: 10.3390/polym11111782 (PMC6918325; doi:10.3390/polym11111782)
Supplement: Supplementary file 1 [file polymers-11-01782-s001.pdf]

Supporting Information

Construction of antifouling membrane surfaces through layer-by-layer self-assembly of lignosulfonate and polyethyleneimine

Lin Gu[1], Meng-Yun Xie[2], Yu Jin[2], Min He[2], Xiao-Yan Xing[2], Yuan Yu[2], Qing-Yun Wu[1,2*]

[1] *School of Chemical Engineering and Technology, Sun Yat-sen University, Zhuhai, 519082, China*

[2] *Faculty of Materials Science and Chemical Engineering, Ningbo University, Ningbo 315211, P.R. China*

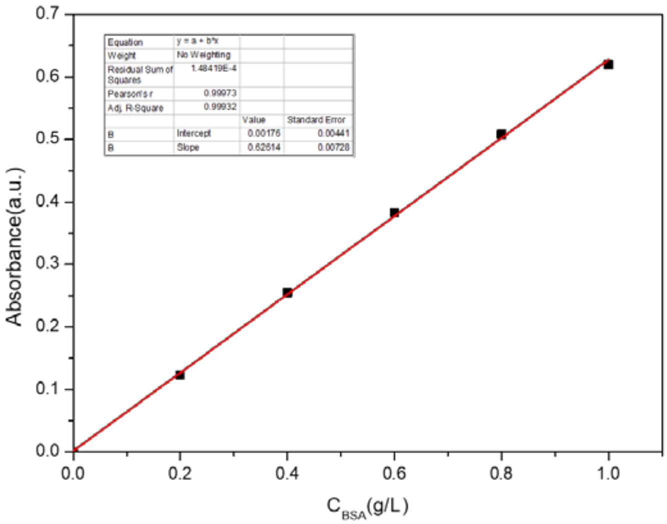

Figure S1. A standard curve of UV absorbance-BSA concentration.

*Corresponding authors. E-mail: wuqingyun@nbu.edu.cn (Q.-Y. Wu)

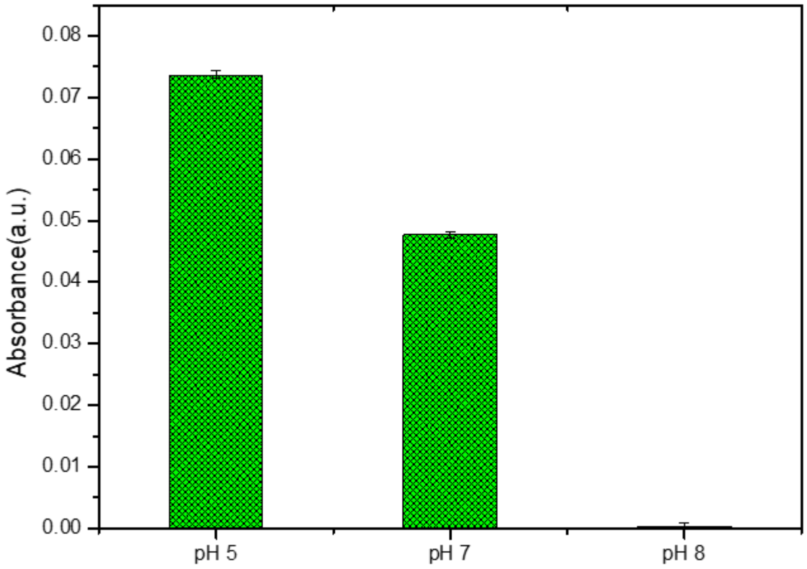

Figure S2. UV absorbance at 280 nm of aqueous solutions after immersing the PSf-5BL membranes prepared at different pH values for 2 h.

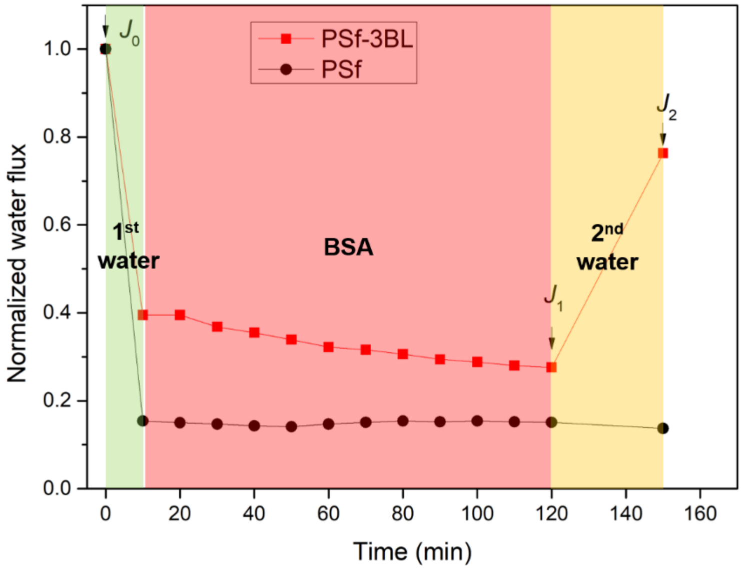

Figure S3. Flux of water and BSA: first for pure water, second for BSA and third for pure water after washing the membranes for 30 min.
